# Dynamic micro-organization of P2X7 receptors revealed by PALM based single particle tracking

**DOI:** 10.3389/fncel.2013.00232

**Published:** 2013-11-26

**Authors:** Amulya N. Shrivastava, Pamela C. Rodriguez, Antoine Triller, Marianne Renner

**Affiliations:** INSERM U1024–CNRS 8197, Biologie Cellulaire de la Synapse, Institut de Biologie de l'École Normale SupérieureParis, France

**Keywords:** P2X7 receptors, P2X receptors, ATP, single particle tracking, PALM, diffusion

## Abstract

Adenosine triphosphate (ATP)-gated P2X7 receptors (P2X7Rs) are members of the purinergic receptor family that are expressed in several cell types including neurons. A high concentration of ATP is required for the channel opening of P2X7Rs compared to other members of this receptor family. Recent work suggests that ATP binding to members of the P2X receptor family determines the diffusion and localization of these receptors on the plasma membrane of neurons. Here, we employed single particle tracking photoactivated localization microscopy (sptPALM) to study the diffusion and ATP-dependence of rat P2X7Rs. Dendra2-tagged P2X7Rs were transfected in hippocampal neurons and imaged on proximal dendrites. Our results suggest the presence of two populations of P2X7Rs within the extra-synaptic membrane: a population composed of rapidly diffusing receptors and one stabilized within nanoclusters (~100 nm diameter). P2X7R trajectories were rarely observed at synaptic sites. P2X7R mutations in the ATP-binding site (K64A) or the conserved phosphorylation site (K17A) resulted in faster- and slower-diffusing receptors, respectively. Furthermore, ATP differentially accelerated wild type and K17A-mutant receptors but not K64A-mutant receptors. Our results indicate that receptor conformation plays a critical role in regulating ATP-mediated changes in P2X7R diffusion and micro-organization.

## Introduction

ATP-gated purinergic P2X receptors (P2XRs) form homo- or hetero-trimeric receptors that can be composed of 7 different subunits, P2X1-P2X7. Slow-desensitizing P2X7Rs are unique members of the ATP-gated P2X receptor family with a characteristic long C-terminus (279 amino acids) and display unusually high EC_50_ for ATP (> 100 μM) (Rassendren et al., 1997). P2X7Rs are expressed in both neuronal and non-neuronal cell types. However, the lack of specific antibodies and the existence of multiple splice variants of this receptor make it difficult to predict their expression and localization (Anderson and Nedergaard, 2006; Kaczmarek-Hájek et al., 2012). Recent development of P2X7-EGFP transgenic mice provides evidence of their expression in several brain regions and in both glial and neuronal cells (GENSAT, www.gensat.org). In the hippocampus of EGFP-P2X7R expressing mice, expression can be seen primarily in the dentate gyrus and CA3 region (*Cornu Ammonis*) suggesting a cell-type specific expression (GENSAT, http://www.gensat.org/).

The development of quantum dot based SPT (QD-SPT) studies has changed our understanding of activity-dependent dynamics of neurotransmitter receptors (Dahan et al., 2003; Groc et al., 2004; Ehlers et al., 2007; Lévi et al., 2008; Bannai et al., 2009). Recently, QD-SPT was successfully implemented to study ATP-dependent modulation of P2X receptor dynamics on the plasma membrane of spinal cord neurons (P2X2Rs, Shrivastava et al., 2011a), hippocampal neurons (P2X2Rs, Richler et al., 2011) and microglia (P2X4Rs, Toulme and Khakh, 2012). These studies suggested that calcium-influx through P2XRs along with the conformational changes induced by ATP binding, govern the diffusion of these receptors. A second important outcome of these studies in neurons suggested exclusion of P2X2R trajectories from synaptic areas despite their over-expression. However, a major limitation of QD usage is their large size (10–30 nm), which can restrict their accessibility to narrow spaces including the synaptic cleft. This shortcoming can be circumvented with single particle tracking photoactivated localization microscopy (sptPALM), an approach that makes use of genetically encoded fluorescent proteins.

PALM relies on the stochastic activation of photoactivatable or photoconvertible proteins to obtain super-resolution images. More precisely, using low intensity activation light, a small percentage of fluorescent proteins are activated at a given time-interval. Each fluorophore is then localized with high precision by fitting its fluorescence emission with a two-dimensional Gaussian function (Betzig et al., 2006; Hess et al., 2006). Since each photo-converted molecule remains visible for a short time period before photobleaching, it is possible to track their position allowing the generation of trajectories and the computation of diffusion coefficients. This approach of single particle tracking using PALM (sptPALM) has been recently successfully employed (Manley et al., 2008; Hoze et al., 2012). The stochastic activation of several well-separated molecules over a given time period provides a random sampling with hundreds of trajectories. In addition, rapid photobleaching allows the sampling of a large number of molecules (hundreds to thousands) with higher accuracy within a given field of view.

A high-level of ATP is released during tissue injury, inflammatory pain, and even in some neurodegenerative disorders (Orellana et al., 2011; Shrivastava et al., 2013, reviewed in Khakh and North, 2012). Therefore, it is important to understand ATP-dependent reorganization and clustering of P2X7Rs and identify factors defining their dynamics. More specifically, we aimed to study how ATP regulates the diffusion of P2X7Rs. We performed high-density mapping of P2X7Rs using the sptPALM approach. Even after over-expression and high-density imaging, P2X7Rs were rarely detected at synapses, suggesting a predominantly non-synaptic localization of these receptors and no synaptic enrichment. Consequently, we focused our study on extra-synaptic receptors. We found that non-synaptic P2X7Rs can be either freely diffusing or trapped within nanoclusters. Mutation disturbing the conserved N-terminus phosphorylation site (Boué-Grabot et al., 2000) revealed that the N-terminal conformation regulates P2X7R diffusion. In addition, perturbation of the ATP-binding pocket by point mutation (Wilkinson et al., 2006) or by ATP binding (Hattori and Gouaux, 2012) altered receptor diffusion. Altogether, our results suggest that the structural conformation of P2X7Rs determines their mobility and confinement on the plasma membrane.

## Materials and methods

### Plasmid and antibodies

Rat P2X7R-EGFP plasmid was kindly provided by Francois Rassendran (Montpellier). The EGFP sequence was downstream of P2X7 coding region between *AgeI* and *NotI* restriction sites (pEGFP-N1 vector) and generated as described previously (Compan et al., 2012). Sequence coding for EGFP was replaced with Dendra2 sequence to generate P2X7-Dendra2 plasmid. Dendra2 is a monomeric fluorescent protein that undergoes irreversible photoconversion from green- to red-emitting state and has a high photo-conversion yield (Adam et al., 2009). Site-directed mutagenesis (Agilent) was performed on P2X7-Dendra2 plasmid to generate K17A and K64A mutant P2X7Rs. The following antibodies were used for immunocytochemistry: rabbit Tau (1:1000, Synaptic System), mouse MAP2 (1:1000, Millipore) and rabbit Synapsin (1:800, Synaptic System).

### Cell culture and transfection

Primary hippocampal cultures were prepared from 18-days-old Sprague–Dawley rat embryos. Neurons were plated at a density of 0.6 × 10^5^ cells/well on 18 mm coverslips pre-coated with 80 μg/ml poly-D, L-ornithine (Sigma). Freshly dissociated cells were plated in neuronal attachment media consisting of 10% horse serum (PAA Labs), 1 mM sodium pyruvate (Life Technologies) and 2 mM glutamine (Life Technologies) in MEM (Life Technologies). Three hours after plating, media was replaced with neurobasal media containing 2 mM glutamine and 2% B27. Cells were maintained by replacing one-fourth of the medium with fresh culture medium every week. Transfection was performed using Lipofectamine-2000 reagent (Invitrogen) according to the manufacturer's instructions. Cells were transfected with 0.4 μg plasmid per coverslip at days *in vitro* (DIV) 12. Imaging was performed on DIV 14.

### Immunocytochemistry

Immunocytochemistry was performed following fixation of cells in 4% (w/v) paraformaldehyde (Serva Feinbiochemica, Germany) and permeabilization with 0.1% triton-X. Cells were incubated with primary antibodies against TAU (1:1000), MAP2 (1:1000) or Synapsin (1:800) for 1 h at room temperature. After washing, cells were incubated for 45 min at room temperature with appropriate secondary antibody and mounted on slides with Vectashield (Vector Labs).

### PALM setup and imaging

The PALM setup and imaging conditions used have recently been described in detail (Izeddin et al., 2011). PALM imaging was performed on an inverted Nikon Ti Eclipse microscope equipped with activation (405 nm) and excitation lasers (561 nm). Images were acquired using a 100× objective (N.A. 1.49). Before acquisition, any pre-converted P2X7-Dendra2 fluorescence was bleached using high-intensity excitation laser. Activation laser was maintained at low power to allow good separation of randomly converted molecules. Single-molecule Dendra2 signal was separated with a 561 nm dichroic (Di01-R561-25 × 36) and a 617 nm emission filter (FF01-617/73), expanded through a 1.5× lens in the tube-lens of the microscope. Images were acquired using an Andor iXon EMCCD camera (512 × 512 pixel with pixel size of 16 μm) at a frame rate of 20 ms. The z-position was kept stable using the perfect focus system (Nikon) integrated with the microscope.

For PALM imaging without tracking, cells were incubated with multi-colored beads (TetraSpeck, Invitrogen) to correct drift. Imaging was performed for 20,000 frames at 20 Hz. For sptPALM experiments, beads were not used and imaging was performed for 5000–6000 frames at 50 Hz. Imaging was performed in MEM medium without phenol red (Invitrogen) containing 2% B-27, 2 mM glutamine, 1 mM pyruvate, 33 mM glucose, and 20 mM HEPES.

### Image analysis

Multi-Trace Tracking (MTT) algorithm was used without the tracking feature (Serge et al., 2008; Izeddin et al., 2011) for detection of individual fluorophores. For visualization and generation of pointillist and rendered images, in-house written software for MATLAB was used and has been previously described (Izeddin et al., 2011). The point-spread function of each fluorophore was detected and fitted with a 2D Gaussian distribution. The resulting pointillist image consists of all the detections obtained during the period of acquisition. Rendering was performed on the super-resolution image by superimposing the position coordinates of the detected single molecules using a standard deviation σ that had been previously determined by the localization accuracy of single fluorophores (typically 10 nm).

Tracking of PALM traces and diffusion calculation was based on the mean square displacement (MSD) approach (Saxton and Jacobson, 1997), which has been previously used for QD-based studies (Bannai et al., 2009; Renner et al., 2010). The center of each fluorescent activation was determined by Gaussian fit based on the point spread function of the microscope with a spatial resolution of ~20–50 nm. Activations in a given frame were associated with the maximum likely trajectories estimated on previous frames of the image sequence. The MSD was calculated using $MSD(ndt)={\left(N–n\right)}^{–1}\sum_{i=1}^{N–n}\left[{\left(x_{i+n}–x_{i}\right)}^{2}+{\left(y_{i+n}–y_{i}\right)}^{2}\right]$, where *x*_i_ and *y*_i_ are the coordinates of an object on frame *I*, *N* is the total number of steps in the trajectory, *dt* is the time between two successive frames, and *ndt* is the time interval over which displacement is averaged (Saxton and Jacobson, 1997; Triller and Choquet, 2008). The diffusion coefficient *D* was calculated by fitting the first 2–5 points of the MSD plot vs. time with the equation *MSD*(*t*) = 4*D*_2−5_*t* + 4σ^2^*_x_*, with σ*_x_* as the spot localization accuracy in one direction. Area explored is defined as the total surface area covered by the trajectory divided by the number of steps in the trajectory.

Once trajectories were generated, they were localized on top of synapses or nanoclusters. Diffraction limited FM4-64 images were used to separate synaptic and non-synaptic trajectories. Super-resolved rendered images obtained using the MTT-algorithm, were used to differentiate between free trajectories and those trapped within nanocluster. As a first step, images were filtered by wavelet segmentation using an interface implemented in Metamorph (Racine et al., 2007) to generate background free masks. Trajectories overlapping the thresholded FM4-64 images were termed “synaptic” and those overlapping the thresholded nanoclusters were termed “trapped.”

### Statistics and image preparation

Experiments were performed on 3–4 independent cultures prepared from different animals on different days. Diffusion data presented shows the distribution of diffusion coefficients pooled from all independent experiments. Kolmogorov-Smirnov test was used to test the difference in distribution. Tracking, rendering, and image analysis were performed on Matlab. Images were prepared using Microsoft excel and Graph Pad Prism.

## Results

### Dendritic expression of P2X7 receptors in transfected neurons

**Figure 1:**
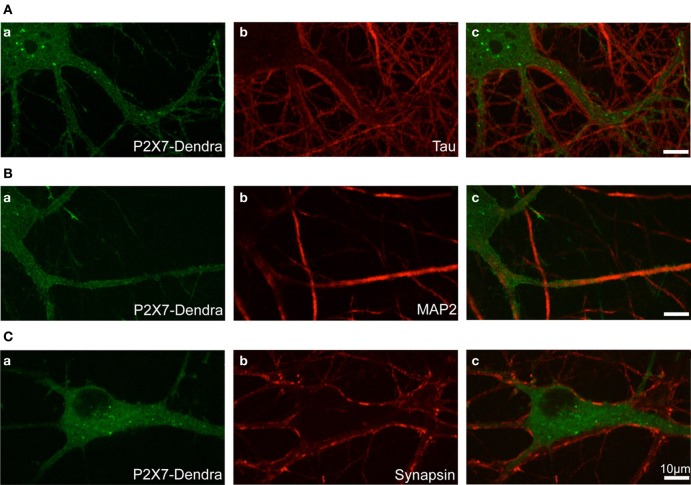
**Localization of transfected P2X7-Dendra2 receptors in dendrites**. Dendra2-tagged P2X7Rs were transfected in hippocampal neurons at DIV 12. Forty-eight hours after transfection, immunocytochemistry was performed to determine the localization of P2X7Rs. **(Aa,Ba,Ca)** Diffused labeling of P2X7Rs with expression in cell body and neurites can be observed. **(Aa–Ac)** Labeling of axons with tau antibody shows axons running apposed to the processes expressing P2X7-Dendra2 receptors. **(Ba–Bc)** MAP2 labeling of dendrites show an overlap with P2X7-Dendra2 expressing neurites. **(Ca–Cc)** Synapse labeling with pre-synaptic synapsin protein shows pre-synaptic boutons are apposed to P2X7-Dendra2 expressing processes.

Photo-convertible Dendra2-tagged P2X7R plasmid was transfected in hippocampal neurons at DIV 12 (days *in vitro*) and experiments performed at DIV 14. To visualize the distribution and localization of P2X7-Dendra2 receptors, we performed immunocytochemistry to label axons (tau), dendrites (MAP2), and synapses (synapsin). P2X7-Dendra2 receptors showed a ubiquitous and uniformly diffused expression in most of the transfected neurons (Figures 1Aa,Ba,Ca). Tau staining revealed axons running at the periphery of processes expressing P2X7-Dendra2 receptors (Figures 1Ab,Ac), suggesting dendritic localization of these receptors. This dendritic localization is confirmed by overlap of P2X7-Dendra2 receptor expressing processes with MAP2 (Figures 1Bb,Bc). Labeling with the pre-synaptic marker synapsin further validated dendritic localization. Synapsin staining apposed P2X7-Dendra2 expressing processes (Figures 1Cb,Cc). These results suggest that transfected P2X7-Dendra2 receptors were targeted to proximal dendrites.

In agreement with previous reports (Díaz-Hernandez et al., 2008), we additionally observed the expression of P2X7Rs in growth cones and fine processes distant from the cell body (not shown), suggesting their localization in distal axons. We focused this work only on proximal dendrites to study post-synaptic P2X7R dynamics.

### Super-resolution PALM imaging reveals nanoclusters of P2X7Rs

**Figure 2:**
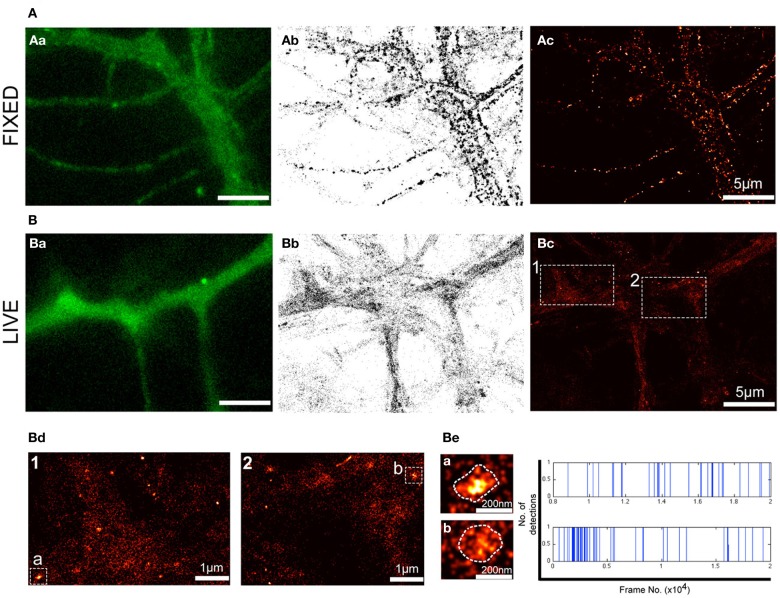
**PALM reveals nanoclusters of P2X7Rs**. PALM imaging was performed on live **(A)** and fixed **(B)** neurons transfected with P2X7-Dendra2. **(Aa,Ba)** Representative fluorescent images of a section of dendrite before PALM imaging was performed. **(Ab,Bb)** Pointillist images displaying all detections obtained for P2X7-Dendra2. **(Ac,Bc)** Super-resolved rendered image generated with a pixel size of 5 nm. Note fixation-induced clustering in **(Ab,Ac)**. **(Bd)** Boxed-region 1 and 2 in **(Bc)** at higher magnification shows predominantly random, but also some nanoclusters of P2X7-Dendra2 receptors. **(Be)** Two random nanoclusters, “cluster a” from box 1 and “cluster b” from box 2, shown at higher magnification. Each detection within clusters “a” and “b” is represented as a blue vertical line and plotted as a function of time (x-axis).

Although fluorescence imaging has considerably accelerated the field of cell biology, the diffraction of light limits its optical resolution and the ability to accurately determine the size of a given structure and the localization of molecules of interest. Even more challenging is the localization of membrane receptors such as P2X7Rs that exhibit a predominantly diffused distribution (Figure 1). To overcome these obstacles, we employed PALM imaging to visualize the distribution of P2X7-Dendra2 receptors at much higher resolution (20–50 nm). PALM was performed on paraformaldehyde fixed (Figure 2A) and live (Figure 2B) neurons transfected with P2X7-Dendra2 receptors. Figures 2Aa,Ba show diffraction limited fluorescent images before PALM acquisition. While the distribution of P2X7Rs appeared unaltered, live cells exhibited much healthier morphology (Figures 2Aa,Ba). PALM was performed by green-to-red photoconversion of Dendra2 for a total of 20,000 frames at 20 Hz. PALM acquisitions are represented as pointillist (each point represents one detection, Figures 2Ab,Bb) or rendered (super-resolved, Figures 2Ac,Bc) images. Visual comparison of pointillist and rendered images of fixed cells (Figures 2Ab,Ac) showed a clustered distribution compared to live cells (Figures 2Bb,Bc). Such large clusters of P2X7-Dendra2 receptors is believed to be a fixation artifact and therefore not suitable for further analysis. Hereafter, we only used live-cell PALM to study P2X7R dynamics.

Notably, in P2X7-Dendra2 transfected live neurons, we observed several sub-micron scaled clusters (hereafter referred to as “nanoclusters”) (Figure 2Bc). Two representative regions (1 and 2) are shown at higher magnification (Figure 2Bd). Additionally, two representative nanoclusters, “a” and “b,” from regions 1 and 2, respectively, are shown (Figures 2Bd,Be). The mean ± SEM (standard error of the mean) area of these nanoclusters was 8718 ± 442 nm^2^. Considering a near circular shape of these nanoclusters, an average diameter of 105 ± 3 nm is approximated. To ascertain if these nanoclusters are real clusters and not generated due to multiple detections of a single fluorophore, we plotted all detections within these nanoclusters during the recording period. Representative traces for the two clusters are shown in Figure 2Be (right panel). Each blue vertical line represents the detection of a fluorophore at a particular period of acquisition (frame number). These traces indicate that detections within these nanoclusters were observed throughout the period of acquisition. Given that Dendra 2 is irreversibly photobleached following its photo-conversion, this suggests the presence of several fluorophores within the clusters.

Notably, the majority of P2X7-Dendra2 receptor detections were not clustered, suggesting a predominant presence as single molecules. The observed nanoclusters appear to be enriched in some dendrites. Altogether, these results suggest that transfected P2X7Rs exist in two populations, one as single molecules and a second stabilized in nanoclusters.

### Single particle tracking of P2X7-dendra2 receptors using PALM

**Figure 3:**
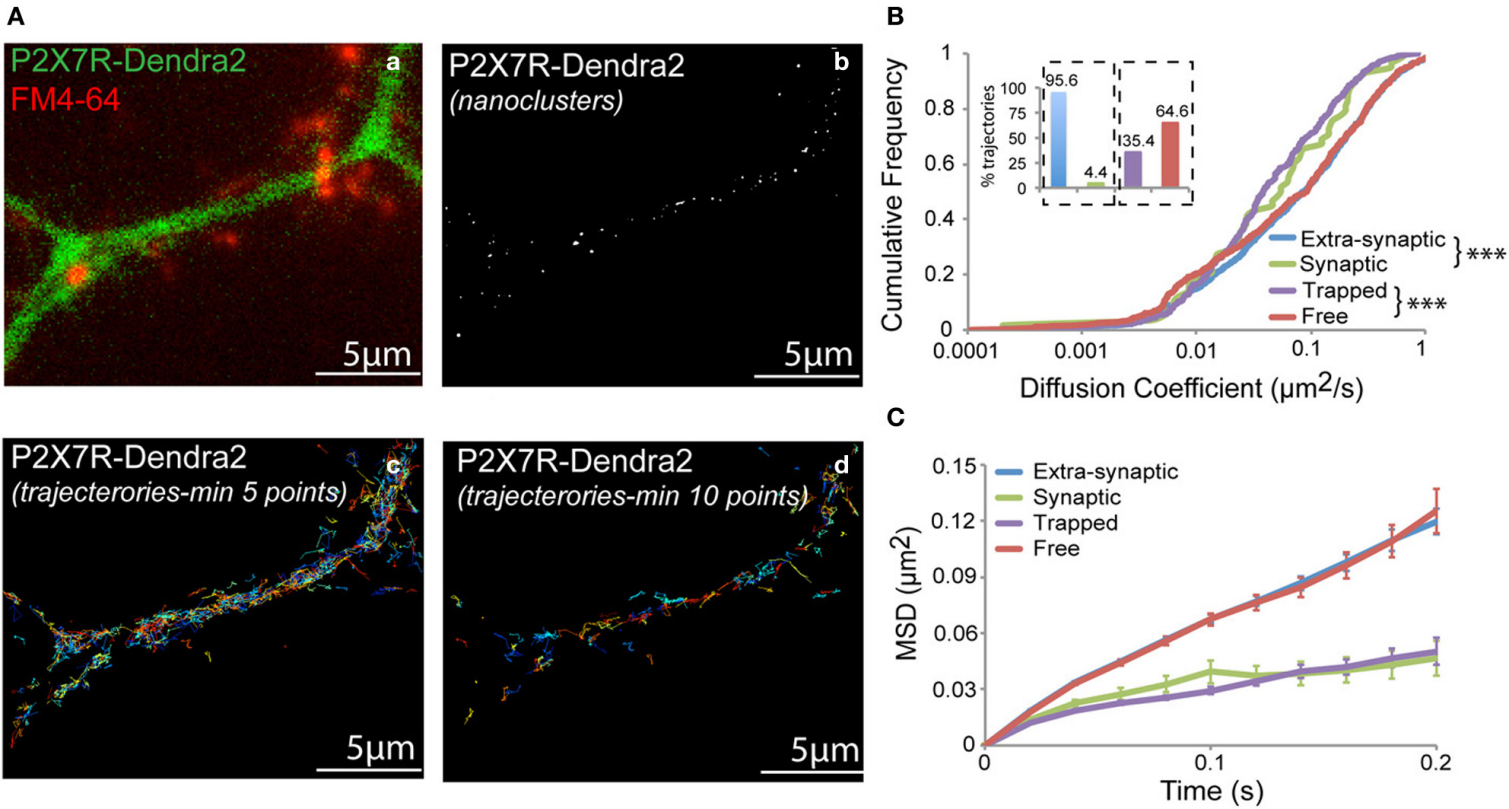
**sptPALM of P2X7-Dendra2 receptors**. Live cell sptPALM was performed at 50 Hz on dendrites expressing P2X7-Dendra2. **(Aa)** Epi-fluorescence of a P2X7-Dendra2 (green) expressing dendrite and FM4-64 (red) labeled synapses. FM4-64 was used for identification of “synaptic” and “extra-synaptic” trajectories. **(Ab)** Representative threshold image obtained from a super-resolved rendered image shows nanoclusters used for localization of trajectories. Trajectories were localized as “trapped” or “free” within nanoclusters. **(Ac,Ad)** Example of trajectories obtained using the sptPALM approach. Minimum 10-point long trajectories were used for diffusion measurements in order to reduce calculation error. **(B)** “Synaptic” and “Trapped” trajectories show slower diffusion compared to “Extra-synaptic” and “Free” trajectories, respectively (Kolmogorov-Smirnov statistical test ^***^p < 0.001). Inset: Proportion of trajectories observed for each fraction (Synaptic + Extra-synaptic = 100% and Trapped + Free = 100%). **(C)** Mean square displacement (MSD) plot shows more confined P2X7Rs at synapses and within nanoclusters (trapped).

Dendra2 undergoes a green-to-red shift in emission wavelength (photo-conversion) when exposed to UV light (405 nm laser). Following photo-conversion, Dendra2 remains switched on for a short time period before irreversibly photobleaching (Adam et al., 2009). This allows the detection and tracking of the fluorophore during the time-period it remains fluorescent. Fast acquisition at a rate of 50 Hz was performed for a short period of 2–3 min. Trajectories for each fluorophore were generated and diffusion properties analyzed. A representative fluorescent image showing a dendrite expressing P2X7-Dendra2 receptors (green) and synapses labeled with FM4-64 (red) is shown in Figure 3Aa. Synaptic labeling allowed us to monitor the mobility of P2X7-Dendra2 receptors within synapses. Threshold-images of nanoclusters obtained from rendered images were used to localize trajectories within these nanoclusters (Figure 3Ab). A distinct advantage of sptPALM is the increased number of trajectories that can be obtained within a short time interval (Figures 3Ac,Ad). Trajectories shown contain a minimum of 5- or 10-detection points. We performed MSD based diffusion analysis to estimate the diffusion coefficient of P2X7-Dendra2 receptors (Saxton and Jacobson, 1997; Triller and Choquet, 2008; Pinaud et al., 2010). To minimize computational error of diffusion coefficients, only trajectories consisting of at least 10-points were considered (Figure 3B). Trajectories of P2X7-Dendra2 receptors were grouped as extra-synaptic (blue) or synaptic (green) according to their localization over FM4-64, and as free (red) if present outside nanoclusters or trapped (purple) if inside nanoclusters. Only 4.4% of the total trajectories were observed at synapses (Figure 3B, inset, blue and green). A low probability of finding trajectories at synapses suggests a preferential extra-synaptic localization of P2X7Rs; thus the distinction between synaptic and extra-synaptic trajectories was not pursued in further experiments. Meanwhile, our data suggested that nearly 35% of the trajectories were localized on nanoclusters (Figure 3B, inset, purple and red). Distributions of diffusion coefficient (D) and MSD of P2X7Rs are plotted in Figures 3B,C. Whereas extra-synaptic (blue) and free (red) trajectories showed similar diffusion coefficients, P2X7-Dendra2 receptor trajectories showed slower diffusion within nanoclusters (purple) and synapses (green). The median diffusion coefficients (μm^2^/s) are: extra-synaptic = 0.077 (*n* = 1246); synaptic = 0.054 (*n* = 59); free = 0.090 (*n* = 597); trapped = 0.038 (*n* = 327).

This difference in the diffusion coefficient is reflected in the shape of the MSD curve (Figure 3C) (Saxton and Jacobson, 1997). Extra-synaptic (blue) and free (red) trajectories showed anomalous diffusion (non-linearly dependent on time) whereas trapped (purple) and synaptic (green) trajectories exhibited relatively confined diffusion. The slow-diffusion and increased confinement within nanoclusters could be due to inter-molecular interactions between P2X7Rs and/or scaffolding molecules localized at these nanoclusters. Thus, sptPALM offers a useful tool to study P2X7 receptor diffusion in and out of nanoclusters formed by these receptors.

### Altered diffusion in K17A and K64A mutant P2X7Rs

**Figure 4:**
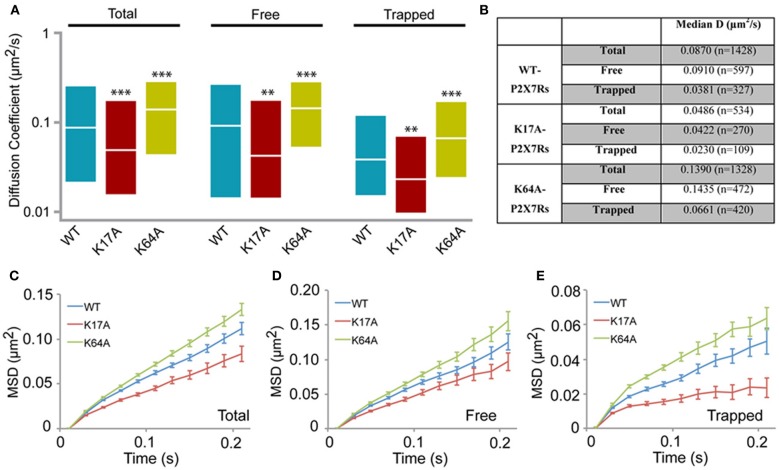
**P2X7R mutants show altered diffusion**. K17A and K64A mutants were generated by sight-directed mutagenesis from the wild-type (WT) P2X7-Dendra2 receptor plasmid. sptPALM was performed and diffusion analyzed. **(A)** Compared to WT-P2X7Rs, K17A-mutant showed slower diffusion, while K64A-mutant showed faster diffusion for total (100%), “free” (~65%) and “trapped” (~35%) population. Plotted data shows a distribution from 25 to 75th percentile and the median value (Kolmogorov-Smirnov statistical test ^***^p < 0.001, ^**^p < 0.01). **(B)** Median diffusion coefficient and number of trajectories for each population. **(C,D,E)** MSD plot shows an increased confined diffusion in K17A-mutant receptors and a decreased confined diffusion in K64A-mutant receptors with respect to wild-type P2X7Rs.

We next generated two mutants to study the role of N-terminus phosphorylation and ATP-binding on P2X7R mobility. Lysine at positions 17 and 64 were substituted with alanine (K17A and K64A). Mutation of lysine at position 17 removes the putative protein kinase-C (PKC) phosphorylation site (15TXK17) without altering receptor gating (Boué-Grabot et al., 2000; Yan et al., 2008). Whereas mutation of lysine-64, found in the ATP-binding site, results in a non-functional P2X7R unable to conduct ions (Wilkinson et al., 2006). Analysis of the distribution of diffusion coefficients as measured by sptPALM revealed a slow diffusion of K17A mutant receptors and a fast diffusion of K64A receptors, relative to WT-P2X7Rs (Figures 4A,B). Notably, these differences in diffusion coefficients of WT and mutant receptors were observed for both free and trapped P2X7Rs. Similarly, MSD curves of the mutants display an opposite effect when compared to WT control. The downward shift of the MSD curve favors a more confined diffusion of K17A-mutant receptor relative to the WT P2X7Rs (red and blue, respectively, Figures 4C–E). Meanwhile, K64A-mutant showed a reduction in confinement as seen by an upward shift of the MSD curve (green and blue, Figures 4C–E). These results suggest that conformation of N-terminus phosphorylation site and ATP-binding site determines P2X7R diffusion behavior on the plasma membrane.

### Modulation of P2X7R diffusion by ATP

**Figure 5:**
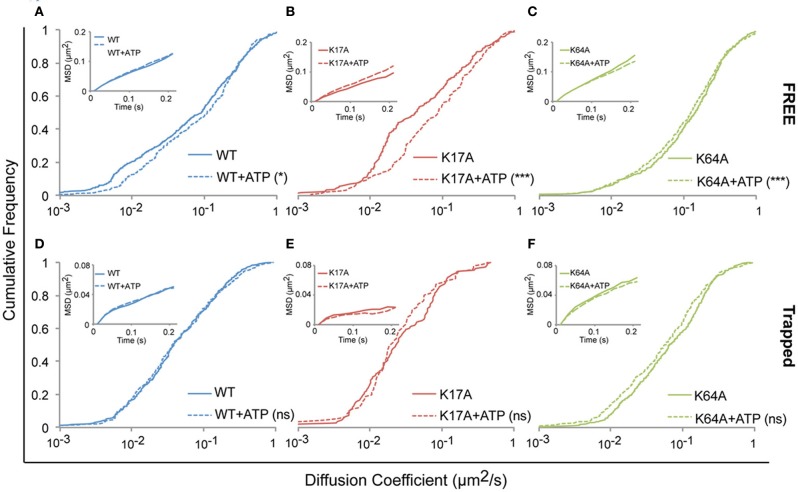
**ATP increased the mobility of P2X7Rs**. Live cell sptPALM was performed either in the absence or presence of ATP (100 μM) in recording medium. (**A–C**, Table 1) ATP differentially increased the diffusion coefficient of “free” WT and K17A-mutant P2X7Rs but not of K64A-mutant receptors. (**D–F**, Table 1) No change in the diffusion coefficient of “trapped” receptors was observed for WT and mutant-P2X7Rs. Inset **(A–F)** shows no change in MSD under any condition. (Kolmogorov-Smirnov test. ^***^p < 0.001, ^*^p < 0.05). Median diffusion coefficient and number of trajectories are shown in Table 1.

We next investigated whether ATP-binding alone could modify WT or mutated P2X7R mobility. One hundred micromolar (100 μM) ATP was added to the recording medium prior to imaging. Changes in diffusion coefficient were then measured for free and trapped populations. ATP-treated cells showed a small but significant increase in the diffusion coefficient of the free population of WT-P2X7Rs (Figure 5A, Table 1). While ATP-dependent acceleration was more pronounced for K17A-mutant P2X7Rs (Figure 5B, Table 1), ATP-binding site K64A mutant, showed no change in mobility for freely diffusing receptors (Figure 5C, Table 1). Notably, ATP had no effect on receptor mobility of trapped receptors (Figures 5D–F, Table 1). MSD plots also displayed no change in P2X7R confinement following ATP-treatment for both “free” and “trapped” receptors (Inset, Figures 5A–F).

**Table 1:** **(Supporting Figure 5) Median diffusion coefficient (μm^2^/s) and number of trajectories (>10 points) used for diffusion calculation**.

		**Control median *D* (μm**^2^**/s**	**+ATP median *D* (μm**^2^**/s)**
WT-P2X7Rs	Non-nanoclusters	0.0910 (*n* = 597)	0.1087 (*n* = 403)*
	Nanoclusters	0.0381 (*n* = 327)	0.0367 (*n* = 365)^ns^
K17A-P2X7Rs	Non-nanoclusters	0.0422 (*n* = 270)	0.0855 (*n* = 143)***
	Nanoclusters	0.0230 (*n* = 109)	0.0192 (*n* = 92)^ns^
K64A-P2X7Rs	Non-nanoclusters	0.1435 (*n* = 472)	0.1247 (*n* = 504)^ns^
	Nanoclusters	0.0661 (*n* = 420)	0.0570 (*n* = 327)^ns^

Kolmogorov-Smirnov test between Control and ATP treated cells.

**Figure 6:**
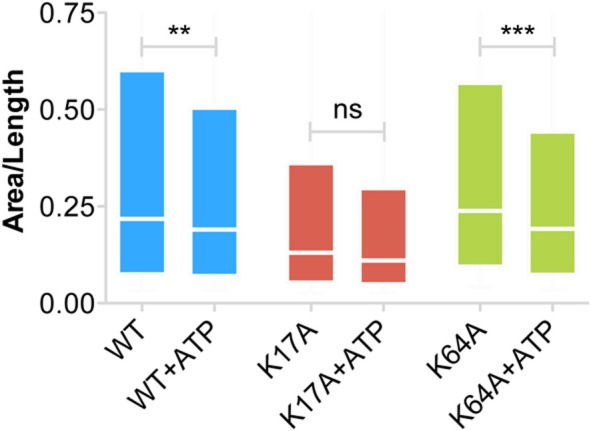
**Increased confinement of P2X7Rs trapped within nanoclusters**. Area (pixels) per unit length of trajectory (number of steps) provides a good estimate of the area explored by trajectories and thus the size of the confinement domain. WT- and K64A-PX7Rs “trapped” within nanoclusters showed a reduction in the area explored or increased confinement in the presence of ATP. Graph shows a distribution from 25th percentile to 75th percentile. (Kolmogorov-Smirnov test. ^***^p < 0.001, ^**^p < 0.01, ns, no significant difference).

Our inability to observe any change in diffusion coefficient and MSD of trapped P2X7Rs suggests that ATP does not affect this population of receptors. However, as the error in MSD calculation is higher for small length trajectories, we also looked at the surface area explored per unit length (length = number of time steps in the trajectory) of P2X7R trajectories that were trapped in nanoclusters. This parameter is independent of the length of the trajectories and gives a good estimate of receptor confinement. We observed a reduction in the area explored/length for both WT and mutant receptors (Figure 6). The median area/length (in pixels) was determined to be: *WT* = 0.2175 (*n* = 2384), *WT* + *ATP* = 0.1902 (*n* = 2457), K17A = 0.1305 (*n* = 816), K17A + ATP = 0.1104 (*n* = 556), K64A = 0.2387 (*n* = 2911), K64A + *ATP* = 0.1923 (*n* = 2225). The above data was obtained from trajectories with a minimum length composed of 5 detections. A similar reduction in area explored was observed with a minimum of 10-point trajectories. These observations suggest that receptors “trapped” within nanoclusters exhibit increased confinement in the presence of extracellular ATP. Notably, ATP had the highest effect on K64A-mutant receptors, which exhibit a disruption in the ATP-binding site (Figure 6). This suggests that direct binding of ATP has no role in mediating change in confinement within nanoclusters.

## Discussion

### Non-synaptic nanoclusters of P2X7Rs

Previous studies using QD based SPT suggested that members of the P2XR family are not enriched at synapses (Richler et al., 2011; Shrivastava et al., 2011a). Weak synaptic labeling of P2XRs could be due to weak expression of these receptors within the synapse or due to the inaccessibility of QD-antibody complexes to penetrate the synaptic cleft. To avoid size-bias of QD-labeling, we employed super-resolution PALM imaging to study the localization and dynamics of synaptic P2X7Rs. Following transfection of P2X7R plasmid C-terminally tagged with photo-convertible Dendra2 protein, we could identify two different populations of P2X7Rs: freely diffusing and trapped as nanoclusters. However, neither of the two populations of P2X7Rs were observed at FM4-64 labeled synapses, supporting previous studies. Thus, it appears that members of the P2XR family are predominantly non-synaptic irrespective of cell type. Further experiments are needed to see if maturation of synapses and cell-types may contribute to the formation of nanoclusters.

A main drawback of PALM is the use of transfection, which may induce over-expression and modifications in the localization of proteins and receptors. Unfortunately, the absence of any specific antibodies suitable for single molecule imaging precludes the study on endogenous receptors. In any case, even tough we cannot completely rule out that nanoclusters of P2X7Rs could be due to over-expression, a number of observations point toward their existence. First, the difference in diffusion coefficient and confinement of WT and P2X7R mutants within nanoclusters suggests conformation-specific dynamics within these nanoclusters. Receptor aggregates are not expected to exhibit such behavior. Second, nanoclusters were rarely observed at synapses irrespective of synapse density, favoring non-random occurrence. Third, ATP modified the dynamics of molecules within the nanoclusters, suggesting an activity-dependent regulation within these nanodomains and indicating that there are signaling-related mechanisms that can modify the internal organization of nanoclusters. Therefore, such nanoclusters of P2X7Rs, possibly act as a signaling platform for P2X7Rs. This points toward the presence of P2X7R (or P2XR) interacting molecules, which may induce clustering at non-synaptic localizations. In fact, we observed non-synaptic clusters of P2X2R-subtype in spinal cord neurons supporting the existence of such receptor-stabilizing nanoclusters (Shrivastava et al., 2011a). In the same study, we found that ~20% of these non-synaptic P2X2Rs were associated with GABA_A_Rs. Work by another group also observed that non-synaptic P2X4Rs were associated with GABA_A_Rs (Jo et al., 2011). Such non-synaptic localization of members of P2XRs, along with their ability to interact with several cys-loop receptors, suggest that P2XRs may be stabilized by their interaction with cys-loop receptors (Khakh et al., 2000, 2005; Sokolova et al., 2001; Boué-Grabot et al., 2004a,b; Shrivastava et al., 2011a, reviewed in Shrivastava et al., 2011b). Notably, no cross talk involving P2X7Rs has been reported. In addition to neurotransmitter receptors (e.g., GABA_A_Rs), P2XR-interacting proteins (reviewed in Kaczmarek-Hájek et al., 2012) and lipid rafts could also contribute to the clustering of P2X7Rs. Biochemical evidence suggests that P2X7Rs can be associated with lipid rafts (Garcia-Marcos et al., 2006; Barth et al., 2007; Gonnord et al., 2009) and could therefore provide a platform where P2X7Rs can cluster.

### Dynamics of P2X7Rs

The majority of P2X7Rs were observed as freely diffusing receptors within the extra-synaptic space. Even after overexpression and high-density sampling by sptPALM, we rarely observed P2X7R trajectories at synapses, suggesting their preference for extra-synaptic localization without any synaptic enrichment. Though nearly 65% of the trajectories were observed as free diffusing, we do not rule out that a small proportion of such free-trajectories could be due to saturation of the capacity of P2X7-clustering protein(s), resulting in over-spill of single P2X7Rs. Compared to WT-P2X7Rs, the diffusion of P2X7Rs lacking the putative PKC phosphorylation site (K17A-mutated) was more confined. Phosphorylation-dependent change in receptor conformation could be a possible explanation for the slower diffusion of K17A-mutant. However, recent work argues against this possibility. It has been reported that mutation of threonine within the conserved PKC site (15TXK17) resulted in a more sensitive agonist response. Contrarily, mutation of K-17 did not alter receptor gating, even though the phosphorylation site was removed (Yan et al., 2008, 2010). This suggests that phosphorylation itself is not a pre-requisite for P2X7R gating and it is the N-terminus structure that may shape P2X7R channel properties, including diffusion. Recent work from our laboratory showed that PKC-phosphorylation of glycine receptor reduced its binding affinity to the scaffold molecule gephyrin, thereby directly determining the amount of receptors at inhibitory synapses (Specht et al., 2011). Although unrelated to glycine receptors, a PKC phosphorylation-dependent interaction of P2XRs with scaffold proteins or other receptors might contribute to their stability in the plasma membrane nanodomains. More work is needed to further understand how phosphorylation-state of P2XRs contributes to their diffusion, localization and interaction with other proteins.

Under physiological conditions, WT-P2X7Rs are composed of both “closed” and “open” receptors depending on the level of ATP (Yan et al., 2010). Previous studies based on the crystal structure of P2X4Rs showed significant structural differences between the ATP-bound (open) and unbound (closed) receptors (Kawate et al., 2009; Hattori and Gouaux, 2012). A mutation (K64A) within the ATP-binding pocket renders P2X7Rs non-functional and unresponsive to ATP (Jiang et al., 2000; Wilkinson et al., 2006). However, we found that the K64A-P2X7R mutant exhibited a faster diffusion compared to WT-P2X7Rs. Since the K64A-mutated receptors lack the ATP binding site, they are preferentially present in a “closed” conformation. Moreover as the sptPALM experiments were performed in an ATP-free medium, most WT P2X7Rs were also expected to be present in a closed conformation. Therefore, the faster diffusion of K64A compared to WT-P2X7Rs is likely due to differences in receptor conformation within the ATP-biding region caused by the point mutation.

In order to determine whether other perturbations (e.g., opening and closing) of the ATP-binding pocket also affect the diffusion coefficient of P2X7Rs, exogenous ATP was added to the imaging media. Following addition of ATP, an increase in the mobility was observed for WT- and K17A- but not for K64A-P2X7Rs. These results further support that perturbation of the ATP-binding pocket through either a point mutation (K64A) or through the opening of receptors after addition of ATP, causes a change in the diffusion coefficient of P2X7Rs. Consequently, ATP-dependent P2X7R conformation regulates its diffusion. It is likely that structural rearrangement within the extracellular ATP-binding pocket leads to changes in conformation of intracellular N- and/or C-terminus, thus modifying diffusion behavior. It should be emphasized that several other factors may contribute to the observed changes in diffusion such as basal phosphorylation state, interaction with scaffold, maturation of neurons, cell-type, network activity, etc. More work is needed to further understand the mechanism of such regulation.

ATP also had an effect on trapped receptors. Both WT and K64A-P2X7Rs trapped in nanoclusters exhibited a decrease in the area explored following ATP treatment. Thus ATP indirectly stabilizes P2X7Rs present within nanoclusters. Indeed, more work is needed to identify the mechanism, but involvement of other endogenous P2XR subtypes cannot be ruled out. ATP-dependent regulation of P2XR mobility and clustering has been demonstrated by several studies (Richler et al., 2011; Shrivastava et al., 2011a; Toulme and Khakh, 2012). We previously reported an ATP-dependent increase in the confinement of P2X2Rs in spinal cord neurons without any observable change in receptor diffusion coefficient (Shrivastava et al., 2011a). However, other studies reported an increase in diffusion coefficient along with reduced confinement following ATP-treatment of P2X2Rs in hippocampal neurons and P2X4Rs in microglia (Richler et al., 2011; Toulme and Khakh, 2012). While the latter work investigated an immediate (~30 s) effect on P2XR diffusion following ATP-application, our work on P2X2Rs in spinal cord neurons looked at the response within 30 min of ATP application. In fact, under similar experimental conditions as those used in our previous work, here we observed only a marginal acceleration of freely diffusing P2X7Rs but an increased confinement of nanocluster-trapped P2X7Rs. It cannot be excluded that these minor variations in the diffusion behavior of P2XR-subtypes could result from intrinsic properties of the receptors and/or depend on cell-types and local calcium concentration.

Altogether, these results suggest a conserved mechanism for the control of P2XR diffusion dynamic, where the binding of ATP on P2XRs initially results in an increased mobility that is eventually followed by slow-down and/or clustering. This hypothesis is strengthened by two additional observations: first, a competitive antagonist, TNP-ATP (2′, 3′-O-(2,4,6-trinitrophenyl) adenosine 5′-triphosphate) that binds the same site as ATP was found to increase clustering of P2X2Rs (Shrivastava et al., 2011a). Second, ATP-induced the formation of hot spots of P2X2-EGFP receptors in transfected neurons (Khakh et al., 2001). Thus, ATP-dependent conformation of P2XRs determines their mobility and clustering on the plasma membrane. An increased level of ATP has been observed in neuro-inflammatory conditions such as neuropathic pain, as well as in neurodegenerative disorders including Alzheimer's disease (Orellana et al., 2011; reviewed in Khakh and North, 2012; Shrivastava et al., 2013). We recently observed that ATP-dependent activation of P2XRs indirectly contributes to the pathogenicity in Alzheimer's disease (Shrivastava et al., 2013). Rapid reorganization of P2XRs following ATP-sensing may create P2XR-clusters that can contribute to neuronal dysfunction by enhancing local calcium-influx in cells. Further work on P2XR dynamics and their nano-organization will shed new light on the involvement of ATP-receptors in physiopathology.

## Author contribiution

Amulya N. Shrivastava, Pamela C. Rodriguez, Marianne Renner, and Antoine Triller designed the experiments and wrote the manuscript. Amulya N. Shrivastava and Pamela C. Rodriguez performed the experiments. Marianne Renner developed the tools for analysis of sptPALM data.

### Conflict of interest statement

The authors declare that the research was conducted in the absence of any commercial or financial relationships that could be construed as a potential conflict of interest.
